# Behavioral flexibility and its drivers in semi-urban vervet monkeys

**DOI:** 10.1093/beheco/arag070

**Published:** 2026-06-13

**Authors:** Paige Barnes, Benjamin Robira, Stéphanie Mercier, Sofia Forss

**Affiliations:** Department of Evolutionary Biology and Environmental Studies, University of Zürich, Winterthurerstrasse 190, 8057 Zürich, Switzerland; Department of Evolutionary Biology and Environmental Studies, University of Zürich, Winterthurerstrasse 190, 8057 Zürich, Switzerland; Department of Evolutionary Biology and Environmental Studies, University of Zürich, Winterthurerstrasse 190, 8057 Zürich, Switzerland; School of Life Sciences, University of KwaZulu-Natal, Private Bag X01, Scottsville, 3209 Pietermaritzburg, South Africa; Inkawu Vervet Project, Mawana Game Reserve, 3115 KwaZulu-Natal, South Africa; Department of Evolutionary Biology and Environmental Studies, University of Zürich, Winterthurerstrasse 190, 8057 Zürich, Switzerland; School of Life Sciences, University of KwaZulu-Natal, Private Bag X01, Scottsville, 3209 Pietermaritzburg, South Africa

**Keywords:** innovativeness, problem-solving, physical cognition, urbanization, nonhuman primates

## Abstract

Phylogenetic comparisons suggest that behavioral flexibility facilitates success in urban environments. It remains less clear whether urbanization fosters cognitive skills that require flexibility, or whether successful individuals in urban environments simply apply pre-evolved skills to solve new problems. To investigate whether variation in anthropogenic experience drives behavioral flexibility required to solve a new technical problem, we presented 42 semi-urban vervet monkeys (*Chlorocebus pygerythrus*) with a 3-phase foraging experiment. Each monkey's tendency to forage on human structures for food was measured to assess their exposure to human environments. We then used a sequential decision-tree-based probabilistic model to describe the series of monkeys’ actions when attempting the task, quantifying 3 behavioral flexibility traits: switch tendency (shifting between known solutions between trials), innovativeness (ability to use unknown solutions), and learning sensitivity (ability to repeat known solutions). We found that individuals' switch tendency and innovativeness were not explained by anthropogenic experience and that the 3 traits were mostly unrelated to each other. Moreover, neither switch tendency, nor innovativeness predicted monkeys’ human food consumption in this habitat. Thus, our study suggests that behavioral flexibility is not driven by anthropogenic foraging experience. Contrasting with the hypothesis that urbanization selects for behavioral flexibility, our findings instead imply that vervet monkeys already had sufficient behavioral flexibility and cognitive capacities to successfully exploit the urban habitat.

## Teaser

Monkeys bring their own skills to the city. Urban animals are often seen as especially flexible or innovative—but do urban environments make them that way? We tested semi-urban vervet monkeys to see if those with more experience entering buildings and trash bins were better at solving a new food puzzle. They were not. Our results suggest vervet monkeys did not need urban life to develop the skills they use to navigate it.

## Introduction

When animals encounter a novel problem, they can either persistently pursue a previously used behavior or exhibit behavioral flexibility. Behavioral flexibility may involve either switching to another known and effective behavior (ie, *switch tendency*; Chow et al. 2016) or modifying the repertoire resulting in a new behavior (ie, *innovativeness*; Kummer and Goodall 1985; Reader and Laland 2003; but see Bandini and Harrison 2020). Under changing conditions, switch tendency and innovativeness can be adaptive (Reader and Laland 2003; Ramsey et al. 2007; Tebbich et al. 2016). Effective switching or innovating requires animals to act or build upon their repertoire of useful behaviors, highlighting the role of cognitive processes such as memory, learning, and decision-making (ie, captured by the term *learning sensitivity*; Chow et al. 2017; Mazza et al. 2018).

On an evolutionary timescale, urban environments represent novel habitats. Phylogenetic comparisons show that behaviorally flexible species are better able to cope with anthropogenic challenges, making them overall more likely to thrive in urban habitats (Lefebvre 1995; Diquelou et al. 2016; Griffin et al. 2017; Wong and Candolin 2024). However, given the evolutionary recency of urbanization, there is limited evidence of adaptation to these environments. In fact, some studies (eg, Caizergues et al. 2022), find little to no signs of behavioral adaptive selection. Therefore, while flexibility may support persistence in urban environments, it is unclear whether urban life selects for cognitive traits or merely draws upon existing behavioral repertoires. While interspecific comparisons demonstrate that flexible species are more likely to persist in urban landscapes, it questions whether urban experience in turn shapes the cognitive traits of individuals.

Within-species comparisons along the urban-rural gradient have used experimental foraging tasks to specifically quantify behavioral flexibility and test its evolutionary relevance in urban environments, but these have revealed contrasting results. Urban individuals sometimes appeared more innovative (field mice, *Apodemus agrarius*: Mazza and Guenther 2021; bullfinches, *Loxigilla barbadensis*: Audet et al. 2016; great tits, *Parus major*: Preiszner et al. 2017), while other findings report equal or lower innovativeness compared with their rural counterparts (foxes, *Vulpes vulpes*: Morton et al. 2023; gray squirrels, *Sciurus carolinensis*: Chow et al. 2021; spotted hyenas, *Crocuta crocuta*: Johnson-Ulrich et al. 2021; multiple bird species: Kark et al. 2007; Papp et al. 2015).

Such contrasting results must, however, be considered cautiously. First, what constitutes an “innovation” varies widely across the literature, both in formal definitions and in observed behaviors (Reader and Laland 2003; van Schaik et al. 2016; Bandini and Harrison 2020). In practice, it is often difficult to distinguish truly novel behaviors from modifications or variants of existing ones, particularly in experimental foraging contexts. Here, we adopt an operational definition of innovativeness as the production of new solutions within the constraints of the task, allowing for comparisons across individuals under standardized conditions. Second, the absence of evident differences in urban and rural innovativeness is not evidence of absence of differences. Indeed, behaviors may be latent and only expressed when required (Masi 2011; Tennie et al. 2020). Apparent urban and rural differences in cognitive performance may reflect variation in task engagement or motivation, rather than differences in cognitive capacities (Sol et al. 2012; van Horik and Madden 2016; Forss et al. 2022). As such, these inconsistent findings from urban-rural comparisons highlight the need to examine how individual variation in anthropogenic experience relates to behavioral flexibility.

Whilst we know that, within populations, individuals’ social and physical environmental experiences, such as predation pressures, food availability, contact with humans, and enrichment, can impact cognitive phenotypes (Call and Tomasello 1996; Damerius et al. 2017; Boogert et al. 2018), less is known about how anthropogenic experiences, such as exploiting urban food sources, affect individuals’ cognitive skills. If urban environments promote behavioral flexibility, we expect individuals with greater exposure to anthropogenic foraging opportunities, often requiring manipulation of anthropogenic structures (Klump et al. 2022), to show enhanced problem-solving abilities. On the contrary, if urban environments do not promote the cognitive skills necessary to flexibly solve novel problems, this would suggest that the species’ pre-existing flexibility and latent cognitive capacity is sufficient for survival, and consequently, individual variation in cognitive abilities stems from other drivers.

Vervet monkeys are an ideal study species to test the cognitive impacts of urbanization, as they are known to be habitat and food generalists and able to adopt flexible foraging strategies (Cheney and Seyfarth 1990; van de Waal and Whiten 2012; Canteloup et al. 2020; Thatcher et al. 2020). Accordingly, part of their successful coping with anthropogenic disturbance could be associated with the species’ ability to profit from human food resources (Patterson et al. 2018; Thatcher et al. 2019). In this study, we investigated whether behavioral flexibility emerges from exposure to urban foraging experiences in a semi-urban vervet monkey (*Chlorocebus pygerythrus*) population living in the Simbithi Eco-Estate in South Africa.

To quantify behavioral flexibility, we focused on 3 complementary components of behavioral flexibility. *Switch tendency* describes an individual's propensity to abandon a previously used solution in favor of an alternative when multiple options are available. *Innovativeness* refers to the ability to generate and successfully implement a solution that the individual has not previously used within the experimental paradigm. *Learning sensitivity* captures the tendency to repeat behaviors that have previously been successful, reflecting the use of past experience to guide current decision-making. Together, these traits describe how individuals flexibly explore, modify, and exploit their behavioral repertoire when facing novel or changing foraging challenges.

Switch tendency, innovativeness, and learning sensitivity are all expected to be advantageous in urbanized environments. Thus, these traits may also be driving the monkeys’ success in this habitat. Consequently, covariation among the 3 traits would imply that selection may have favored a set of adaptive traits forming a so-called “syndrome” (Sih and Del Giudice 2012; Guenther and Brust 2017), as observed for other adaptive trait clusters like aggression-boldness (Biro and Stamps 2008). Selection in urban habitats may favor such an adaptive behavioral flexibility syndrome if the 3 traits jointly enhance foraging success and reproductive output. Alternatively, urban pressures may favor only specific flexibility components at the cost of another, such as high innovativeness and learning sensitivity, but persistence instead of high switch tendency, resulting in tradeoffs between traits (Ducatez et al. 2015).

Consequently, we investigated 3 questions: (i) Does anthropogenic foraging (ie, opportunistically entering anthropogenic structures) in this species transfer to individual variation in problem-solving skills (requiring behavioral flexibility)? (ii) Do flexible traits, such as “switch tendency,” “innovativeness” and “learning sensitivity,” co-vary to form a behavioral syndrome? (iii) Does individual variation in behavioral flexibility relate to (a) successful solving of a technical challenge, and (b) increased human food consumption (as a proxy for successful exploitation of the anthropogenic habitat)?

Should behavioral flexibility be key to individual success in urbanized habitats, and already undergone positive selection, we expect (i) that switch tendency and innovativeness are associated with an individual's anthropogenic foraging tendency, (ii) this population of monkeys will show a syndrome representing high switchers, innovative, and sensitive learners, and (iii) high-switching and innovative individuals should (a) perform better in the foraging experiment and (b) consume more human food.

To answer these questions, we presented 42 individually identified monkeys with a 3-phase foraging experiment (puzzle box) of increasing technicality. We used conditional probabilistic modeling to consider the entire sequence of each monkey's interactions with the puzzle box. This approach enabled us to use decision trees to characterize 3 distinct measures of each individual's behavioral flexibility in the experiment: switch tendency (shifting between solutions between trials), innovativeness (opening different options) and learning sensitivity (repeating successful techniques). Finally, we also measured each monkey's success at accessing the food reward.

## Methods

### Study population

We studied 2 groups of semi-urban vervet monkeys (*N* = 42, including 13 adult females, 4 adult males, and 25 juveniles) ranging in Simbithi Eco-Estate in Ballito, KwaZulu Natal province, South Africa. Simbithi Eco-Estate is a residential, gated community with endemic vegetation on forested trails creating a mosaic of forest patches, human-associated vegetation, houses, and impervious surfaces (eg, roads), all of which are used by the vervet monkeys (Patterson et al. 2019). Both groups were habituated to human observers and behaved naturally in the presence of researchers.

### Foraging experiment

#### Experimental set-up

**Figure 1:**
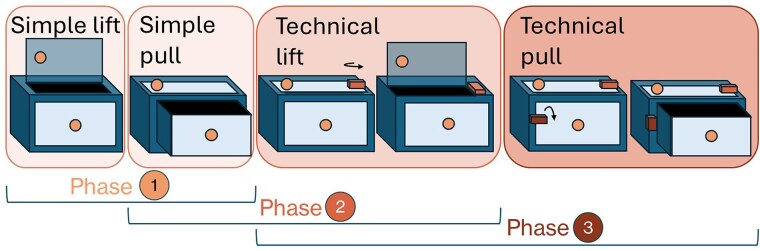
Visualization of the 2 options for each of the 3 phases of the experiment. The colors of the phase and box illustrations correspond to the technicality of the phase due to added opening obstacles along the experimentation, with the darker the graphic illustration, the more technical. Phase 1: the monkey can simply either lift (“simple lift” solution) or pull (“simple pull” solution); phase 2: the monkey can either use the simple pull or unlock a small block at the side of the lid and lift (“technical lift” solution); phase 3: the monkey can either use the technical lift or technical pull solution (incorporating first unlocking a block at the side of the lid and/or at the side of the drawer).

The foraging experiment featured a puzzle box with a 2-option design (Canteloup et al. 2020). This transparent plastic box had the back, bottom, and adjacent sides painted in blue (dimensions: 13 × 10.5 × 7.9 cm) and could be opened in 2 different ways to gain access to a food reward (ie, a peanut) inside: by “lifting” a lid or “pulling” the drawer container (Fig. 1, Figure S1).

Experimental data were collected from September 2023 to January 2024. During this time, we opportunistically presented the monkeys with the 3-phase puzzle box foraging experiment. Experiments were conducted at any time throughout the day from sunrise to sunset. To avoid multiple individuals interacting with the box and inter-individual conflicts, we specifically seized opportunities to target 1 individual and perform the experiment when a limited number of individuals were present. To set up the experiment, we anchored the puzzle box to the ground using camping hooks and placed the box, so it was visible to the target monkey. We changed the direction of the box when moving locations between trials to avoid possible constant directional bias on side manipulation caused by the potential influence of an observer presence on a given side. All experiments were videotaped (see Video coding). We recorded the identity of all monkeys present and their distances during each trial to measure any possible social exposure to the box experiment.

Since these monkeys were naïve to this experimental setup, we first implemented a pre-exposure phase (familiarization phase) to minimize any potential neophobia towards the box, which could impact the likelihood of interacting with it (Ellington et al. 2024). During the familiarization phase, there was no obstacle to accessing the food reward as we removed the top lid and pull drawer of the box and baited it with a peanut on top of the box so that the monkeys could form a positive association to the puzzle box. We ended the familiarization phase once every individual in both groups retrieved a peanut, and all group members were determined to have been within eyeline of at least 1 retrieval. The experiment subsequently comprised 3 phases, each implying a different level of technicality (Fig. 1). Subsequently, in a few situations, we baited the top of the box with an additional peanut to attract the attention to the puzzle box when an individual showed low motivation to participate.

A trial began when a monkey touched the puzzle box and ended when it moved out of arm's length for over a minute (all phases), or it reached a 1-min time limit while within arm's length of the box, interacting or not (phases 2 and 3). Groups were limited to testing up to twice a week on nonconsecutive days to avoid overfeeding and to minimize potential behavioral impacts due to the experiment. An individual was tested a maximum of 5 times a day (mean ± SD: 1.99 ± 1.12; with testing spanning over 97.10 ± 41.71 days) and the time interval between trials was of a minimum of 1 min if unsuccessful, but no delay if successful. If successful, a new peanut was immediately reinserted in the box using the same solution side from the previous trial and thus resetting the box. During this process, the researcher often positioned their body or a hat to block the view of nearby observers. We considered a trial as “successful” when the participant successfully retrieved the peanut, or otherwise as an “attempt” if not.

Following an ethogram, (Supplementary Material, Table S1), we used video records to thoroughly identify whether unsuccessful access to the peanut could be considered as attempts nonetheless (ie, the individual tried to open the box but did not manage to, in contrast to when a monkey ignored or only superficially explored the box). If the interaction with the box was interrupted by a conspecific, we considered this trial as “aborted” and it was not further included in the analyses. All individuals were free to engage in each trial or not (Supplementary Material, Table S2). This means that, for each trial, we considered 4 possible outcomes: successfully lifting, successfully pulling, unsuccessfully lifting, and unsuccessfully pulling. Only unsuccessful lift and pull were nonexclusive, meaning that if an individual attempted a solution (ie, was unsuccessful) at first but was later successful, we only considered the successful event, and the trial could be labeled with the outcome “successful lifting.” However, if the individual attempted both lifting and pulling, the trial would be labeled “unsuccessful lifting and pulling.” Attempts were, however, only counted as single events (ie, we did not consider whether the individual attempted one or several times an opening option within a trial), for the sake of interpretability.

Each phase consisted of several trials (*N*_phase1_ = 444 trials and 41 monkeys (1 monkey never engaged in the experiment), *N*_phase2_ = 174 and 27 monkeys, *N*_phase3_ = 155 and 25 monkeys). While all individuals could participate in phase 1, phases 2 and 3 were constrained to individuals who passed a “phase success” criterion each time. To validate our chosen phase success criterion, the influence of the number of trials per phase on our estimations can be found in the Supplementary Material (Figures S4 to S6).

##### Phase 1—simple lift and simple pull

In phase 1, the lid and the drawer, absent in the familiarization phase, were added, such that the peanut could be retrieved by the simple solutions of either “pulling” the drawer or “lifting” the lid (Fig. 1). We considered that a monkey passed phase 1 when it achieved 10 trial successes in total (not necessarily consecutive successes; consisting of either lifting or pulling, or a mixture of both solutions), as this indicated that the monkey could adequately perform at least 1 simple solution, and this number provided enough trials to robustly examine switch tendencies. Monkeys were free to try the experiment as many times as they wanted but only passed phase 1 if they reached at least 10 successes.

##### Phase 2—technical lift and simple pull

Overall individuals preferred the lift option in phase 1 (83% of all openings and preferred by all but 2 individuals). In phase 2, we increased the technicality of the “lifting” access by adding a twistable lock (a small white block: 2 × 1 × 1 cm) screwed onto the top of the box blocking the lid (Fig. 1). When the block was turned in either direction, the lift solution could be “unlocked”, and the lid could be lifted as in phase 1. Because the monkeys had to use fine motor movements or employ teeth to twist the lock to retrieve the peanut, this was considered a “technical” solution compared with simply lifting and pulling the lid. If the monkeys managed to retrieve the reward within a minute, trials were counted as a success, otherwise as an attempt (if the monkey interacted with the box). Individuals who completed a minimum of 6 trials (not necessarily successes) moved to phase 3. Because individuals differed in their preferred solution type in the initial phase, advancement to subsequent phases was based solely on trial completion criteria rather than on the use or success of a specific solution, ensuring that individuals preferring the pull solution were not systematically excluded.

##### Phase 3—technical lift and technical pull

In phase 3, we maintained the “technical” lift solution from phase 2 and increased the difficulty of the pull solution in the same way as the lift solution (Fig. 1; “technical pull” solution), introducing an equivalent lock on the original pull side. Success and attempts were defined in the same way as phase 2, and phase 3 also ended when the monkeys reached a total of 6 trials. This phase allowed individuals to express technical innovativeness through either solution type, enabling the assessment of innovation independently of initial solution preference.

##### Video coding

All experimental trials were recorded using a Canon camera HDR-CX200 mounted to a tripod, placed on average about 5 m from the box. Individuals’ identities within the eyeline of the box, and distances to the box (ie, within 2, 5, 10, or more than 20 m) were recorded vocally during the filming of the experiment. Trial outcomes were subsequently extracted (Table S1).

### Behavioral observations

We used long-term observational data, where the monkeys were monitored daily from Monday to Friday for up to 8 h between sunrise and sunset, cumulating in 3,137 h of observation time between July 2023 and September 2024. Observers recorded the monkeys’ social and feeding behaviors (Supplementary Material, Table S3) following *ad-libitum* records of specific events (eg, opportunistic feedings on anthropogenic food resources, referred to as anthropogenic foraging tendency) using handheld phones (Blackview BV4900) and CyberTracker version 3.531. Each observer that contributed to this long-term data set passed both individual identification and inter-observer reliability tests which had to be successfully completed prior to collecting data with at least 80% of agreement reached, estimated with a Cohen's kappa test (Supplementary Material, Table S4 and section S5 for further details).

The anthropogenic foraging tendency was defined as each time a monkey entered a building or interacted with a bin. We hypothesized that such instances could accumulate the monkeys’ experiences with anthropogenic artifacts, and thereby promote technical problem-solving skills, useful to innovatively solve a human-made puzzle box. Using *ad-libitum* data, we quantified “anthropogenic foraging tendency” as the number of times individual monkeys entered an anthropogenic structure divided by the total amount of follow hours of that group when that individual was present (range: 3 to 72, mean = 27.8 events per monkey). In addition, we estimated the human food consumption, or the rate at which each individual fed on human food sources by calculating the number of recorded instances an individual was seen to successfully obtain/consume food in the *ad-libitum* data, divided by the corresponding number of observation hours.

### Ethical approvals

This project has been approved by the Animal Research Ethics Committee of the South African University of KwaZulu-Natal (protocol reference number: T20220164), as well as supported by the Environmental Board of Simbithi Eco-Estate in KwaZulu-Natal province, South Africa.

### Statistical analyses

All data processing and analyses were performed with *R* software (v4.4.1; R Core Team 2021).

#### Quantifying individuals’ switch tendency, innovativeness, and learning sensitivity

**Figure 2:**
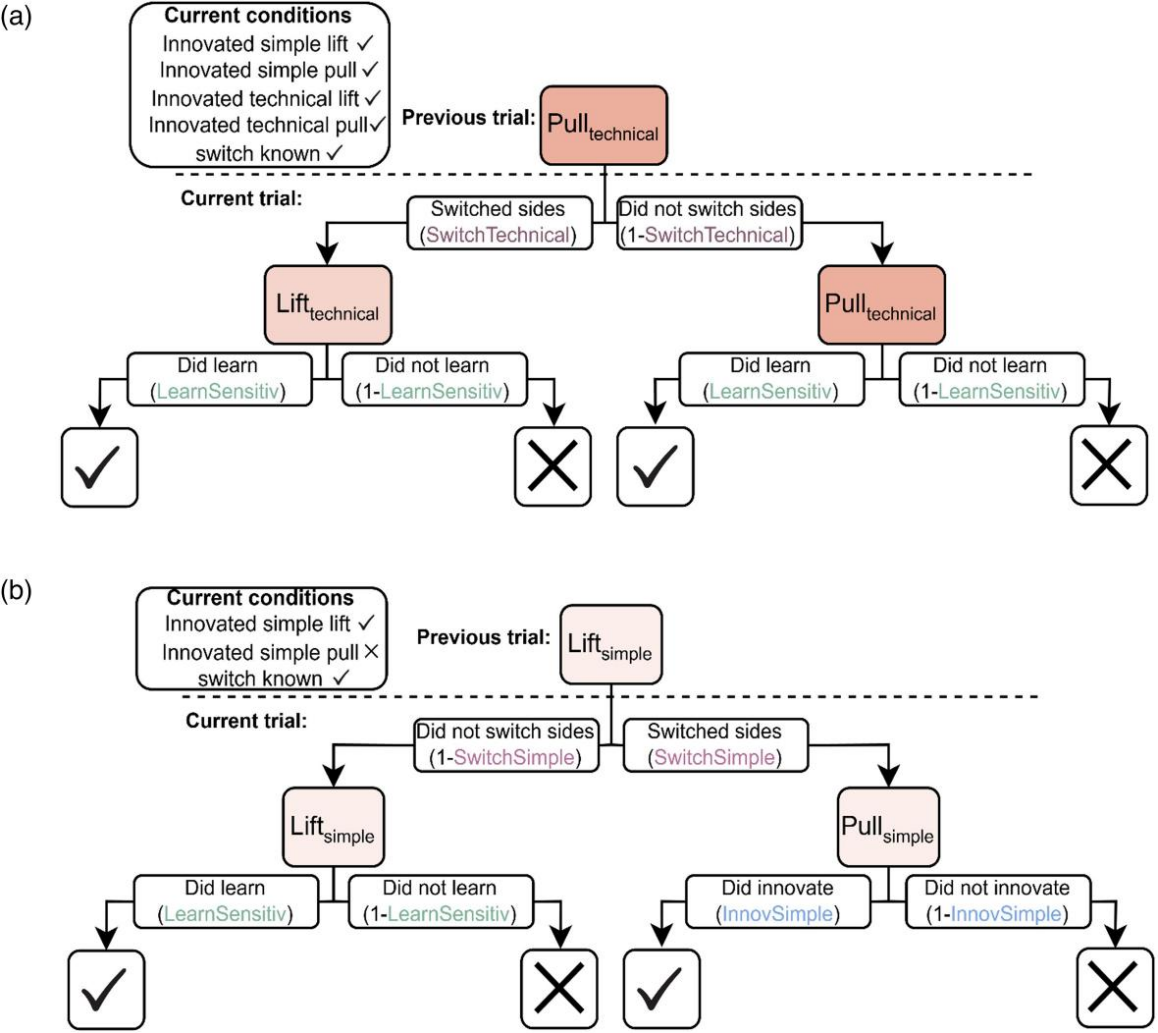
Two examples of decision trees applied to consider the whole temporally ordinated series of each monkey's interactions with the foraging experiment. The top, opaque box indicates the previous trial choice, the second set of opaque boxes indicates which solution option is attempted, and the bottom set of boxes indicates whether the individual successfully opened the box during this trial (a check means it is successful, a cross means it is a failed attempt). Each branch indicates which parameters are updated if this pathway is taken during the trial. The dashed lines show a separation of trials. a) The corresponding tree for a phase 1 trial, knowing that the previous trial solution was a lift, so whether there is a switch in this trial is known (“switch” known), the lift solution is known, and the pull solution is unknown. b) The decision tree corresponding to phase 3 where the previous trial solution was a technical pull solution (“switch” and all solutions are known).

We distinguished “simple” and “technical” challenges (and thus, considered 2 sets of switching and innovative parameters) to disentangle the effects on the cognitive skills when options vary in cognitive and/or motor demand. We used probabilistic modeling to characterize 5 traits for each individual: simple/technical switch tendency, simple/technical innovativeness, and learning sensitivity (Table 1). These scores were calculated using specific decision trees (Fig. 2 and Figure S2) based on the conditions of each trial and updated throughout the entire sequence of trials.

**Table 1:** Estimated parameters for each individual, based on their sequence of trial outcomes, where the simple parameters represent the performance in phase 1, when the challenge is simpler, while the technical solutions represent the traits expressed when the option of at least 1 technical solution is present.

Parameter	Trait	Definition	Phases
*SS*	Simple Switch Tendency	Switch from the previous trial's solution option to alternative option when both are simple. (1-*SS*) if the individual attempts the same solution again.	1
*ST*	Technical Switch Tendency	Switch from the previous trial's solution option to alternative option when at least 1 option is technical. (1-*ST*) if the individual attempts the same solution again.	2, 3
*IS*	Simple Innovativeness	Successfully open the lift or pull for the first time. (1-*IS*) if the individual unsuccessfully attempts the lift or pull solution and has never successfully opened that solution before.	1
*IT*	Technical Innovativeness	Successfully open the technical lift or technical pull for the first time. (1-*IT*) if the individual unsuccessfully attempts the technical lift or technical pull solution and has never successfully opened that solution before.	2, 3
*V*	Learning Sensitivity	Successfully opened a solution that the individual has previously opened. (1-*V*) if the individual unsuccessfully attempts a known solution.	1, 2, 3

We assumed that the probability *P* of observing a certain outcome could depend on 5 parameters constrained between 0 and 1 according to the individuals’ past actions and current phases (Table 1): (i) a switch tendency between known simple solutions (“Switch Simple”, *SS*; in phase 1), (ii) switch tendency when technical solutions are available (“Switch Technical”, *ST*; in phase 2 and 3), (iii) an ability to innovate for the first time one of the simple solutions (“Innovate Simple”, *IS*; in phase 1 and 2), (iv) an ability to innovate for the first time one of the technical solutions (“Innovate Technical”, *IT*; in phase 2 and 3 with technical lift/technical pull) and (v) an ability to reproduce previous performance (ie, a learning sensitivity, *V*). We considered that a monkey switched solutions if they engaged into a solution new for the trial but previously experienced.

We assumed that the monkeys should perform sequential reasoning (Procyk and Joseph 1996), and thus assumed that *P* was the product of 2 components: choosing which solution to attempt (lifting or pulling) and the eventual output of the attempt (innovativeness, if the action was never performed before in previous trials, or learning sensitivity, if the action had already been performed in previous trials). For example, if we assumed that an individual *i* in phase 1 at trial *t* has successfully obtained a peanut by lifting, knowing that it had already lifted in a previous trial but had pulled at trial *t*−1, we would have *P_i,t_* = SS *× V*. This series of decisions, and probabilities of observing a given behavior, can thus be represented by a decision tree (all 45 are shown in the Supplementary Material, Figure S2; Fig. 2 illustrates 2 examples).

For each monkey, we could estimate the likelihood of the observed trial sequence as the product of all the trial outcome probabilities. We estimated which combination of *SS*, *ST*, *IS*, *IT*, and *V* maximized the log-likelihood separately for each monkey using the “optim” function of the *stats* package using the “L-BFGS-B” optimization method to constrain parameter ranges between 0 and 1 (Nash and Varadhan 2011). Best-fit parameters (and their associated 95% confidence intervals) were used to characterize individuals’ switch tendency, innovativeness and learning sensitivity for further analyses and robustness of our inferences (Supplementary Material, sections S7 and S8).

#### Regression modeling and correlations

##### Predicting switch tendency and technical innovativeness

To identify the factors predicting individual variation in switch tendency and technical innovativeness, we modeled individuals’ simple switch tendency scores, *SS*, and technical innovativeness scores, *IT*, as a function of anthropogenic foraging tendency, demographic (a composite measure relative to age, sex and rank, Supplementary Material, section S9) and social composite variables (a composite measure relative to social exposure to the foraging experiment, Supplementary Material, section S9), to reduce model complexity given the low sample size.

The demographic composite variable was derived using principal component analysis (PCA) on individuals’ social rank, sex, and age. Age was coded as a binary variable (juvenile or adult), sex was coded as binary, and rank was determined from agonistic interactions within the group (using Elo-rating scores). All variables were standardized prior to PCA to ensure comparability. The first principal component (PC1), which explained 45.8% of the variance, was used as the demographic composite score in subsequent models. Correlation matrices and PCA visualizations are provided in the Supplementary Material (section S9).

The social composite variable summarized each individual's previous exposure to the foraging experiment. We included 3 standardized measures in the PCA: (i) the number of trials using the same solution within 2 weeks prior to the first successful opening in phase 1 (reflecting prior experience with simple solutions; restricted to 2-week time windows to reflect a relevant memory timescale; see Supplementary Material S11 for sensitivity analyses), (ii) the number of innovation trials observed by the individual prior to starting phase 2 (reflecting exposure to technical innovation), and (iii) the number of phase 2 trials observed within 5 m prior to starting phase 2 (reflecting exposure to the experimental challenge overall). PCA was applied to these variables, and the first principal component (PC1), capturing 42.0% of the variance, was used as the social composite score, with higher values representing greater prior exposure and engagement (Figure S10).

To evaluate the respective influence of anthropogenic foraging tendency, social and demographic variables on an individual's switch tendency, we used a continuous variable (therefore conducting a beta-regression with a logit link function; “glmmTMB” function with the family set to “beta_family” of the *glmmTMB* package; Brooks et al. 2017). For technical innovativeness, due to high heterogeneity, we transformed it into a binary variable (therefore conducting a binomial regression with a logit link function; “glmmTMB” function with the family set to “binomial” of the *glmmTMB* package; Brooks et al. 2017), with “low innovativeness” occurring when the confidence interval around the mean *IT* estimate was lower than 0.5, and “high innovativeness” otherwise (Supplementary Material, Table S7, see Figure S13 for a sensitivity analysis of this cutoff choice). All variables were scaled to a mean of 0 and a standard deviation of 1. We used an information-theoretic approach (Buckland et al. 1997; Anderson and Burnham 2002) to investigate which variable was likely influential. Specifically, we used the sum of the models’ weight in which the variable of interest was included as a proxy to describe the likelihood that this variable influences innovativeness (with respect to other variables considered) and quantified its average estimate (and associated unconditional confidence interval) by computing the shrunk average model (ie, considering all models and their weights; “modavgShrink” function of the *AICcmodavg* package; Mazerolle 2017). We ensured that the most complex model fit was good by (i) verifying that all models converged and were not singular (“check_convergence” and “check_singularity” functions of the *performance* package; Lüdecke et al. 2021), (ii) visually and statistically checking the models’ assumptions (ie, adequate distribution of residuals under the modeled distribution and their homoscedasticity using the *DHARMa* package (Hartig 2024); Supplementary Material, Figures S14 and S15), (iii) verifying the absence of major outlier (“check_outliers” function of the *performance* package; Lüdecke et al. 2021), and (iv) verifying the absence of over-parameterisation (“extractCN” function of the *AICcmodavg* package; Mazerolle 2017); all parameters had low condition values, (Supplementary Material, Figure S14). We also verified that there was no collinearity issue by focusing on the Variance Inflation Factor (VIF < 2, “check_collinearity” function of the *performance* package (Lüdecke et al. 2021), Supplementary Material, Tables S8 and S9).

##### “Behavioral flexibility syndrome”

To investigate the link between multiple traits potentially underlying anthropogenic foraging in vervet monkeys, we evaluated whether the 3 traits *IT, SS,* and *V* formed a behavioral flexibility syndrome (ie, a group of correlated behavioral traits; MacKay and Haskell 2015). To do so, we modeled each variable as a function of the other using beta-regression (as the variables are constrained between 0 and 1; Ferrari and Cribari-Neto 2004) with a logit link (“glmmTMB” function with the family set to “beta_family” of the *glmmTMB* package; Brooks et al. 2017). Prior to fitting, we transformed each output variable *y* such as *y*′ = (*y*(*n −* 1) + 0.5)/*n*, where *n* represents the sample size, to account for values strictly equal to 0 or 1 (Smithson and Verkuilen 2006). As our focus was on individual problem-solving skills that required flexibility, as a measure of innovativeness and switch tendency, we modeled *SS* and *V* as a function of *IT*, and *V* as a function of *SS*.

##### Predicting problem-solving and human food consumption

We first evaluated whether experimental performance links to a monkey's human food consumption in the urban habitat using a Pearson correlation (“cor.test” of the *stats* package; R Core Team 2021).

We then separately investigated the drivers of 2 measures of success (in the foraging experiment and in exploiting human food). For monkeys to successfully retrieve the food reward in phase 3 of the experiment, they were required to use their motor and technical skills, as well as to innovate the solution to the problem presented to them, either innovating the technical lift or technical pull. To avoid circularity in the estimation of the technical innovativeness scores, we excluded phase 3 (where both solutions required technical innovation) from our foraging experiment success calculations. Consequently, performance measures were derived independently of the most technically demanding phase. We tested the effects of individuals’ switch tendency (approximated by *SS*) and innovativeness (approximated by *IT*) on predicting foraging success in phase 1 and 2. We considered the foraging experiment success rate for each individual *i* across the 2 phases, *s_i_* as the weighted average of success rate between the 2 phases such as *s_i_* = (1−*w*)*s_i_*,_1_ + *ws_i_*_,2_ with *w = s*_1_/(*s*_1_  *+ s*_2_) where *s*_1_ and *s*_2_ represent the success rate of all the monkeys tested in phase 1 and 2 respectively. We then modeled the foraging experiment success rate (constrained between 0 and 1) as a function of *IT* and *SS* with a beta-regression (implemented as in 2.4.2.1), considering *SS* with a second-order polynomial form. We used a second-order polynomial (*SS*^2^) based on the reasoning that a monkey could be persistent (low *SS* and thus using same solution over and over) or highly flexible (high *SS*) to successfully retrieve the reward.

To allow for estimate comparability (Schielzeth 2010), both continuous predictors were scaled to a mean of 0 and a standard deviation of 1. We then repeated this procedure with human food consumption as the response variable. We verified the model fit quality as in 2.5.2.1 (Supplementary Material, Tables S10 and S11 and Figures S16 and S17).

## Results

Out of the 42 monkeys from both groups, 41 were initially tested (only one did not engage in the experiment) and 80.5% completed at least 10 trials in phase 1 (33/41, *N*_phase1_ = 444). Of these, 81.8% made it to phase 2 (27/33, *N*_phase2_ = 174) and 92.6% of those individuals made it to phase 3 (25/27, *N*_phase3_ = 155). The number of trials per monkey in phases 1, 2, and 3 were 12.8 (±3.64), 6.40 (±1.03), and 6.5 (±1.27), respectively (when not indicated otherwise, we always refer to a variable by its mean ± SD). For the 3 phases, the overall success rate was 87.2% (387/444), 81.6% (142/174) and 41.3% (64/155), respectively.

Twenty-seven monkeys (65.9%) performed a sufficient number of technical trials to characterize their problem-solving skills in terms of switch tendency between simple (*SS*) and technical solutions (*ST*), innovativeness (either simple *IS* or technical *IT*), and learning sensitivity (*V*). Individuals’ estimates are presented in Figure S12. Overall, switch tendency was relatively low (*SS*: 0.263 ± 0.032; *ST*: 0.469 ± 0.036) and innovativeness varied across technical levels (69.6% and 11.1% with *IS* > 0.5 and *IT* > 0.5, respectively). When the monkeys successfully performed a solution, they were generally good at reproducing it (*V*: 0.792 ± 0.025).

### Anthropogenic experience did not predict innovativeness in urban monkeys

**Figure 3:**
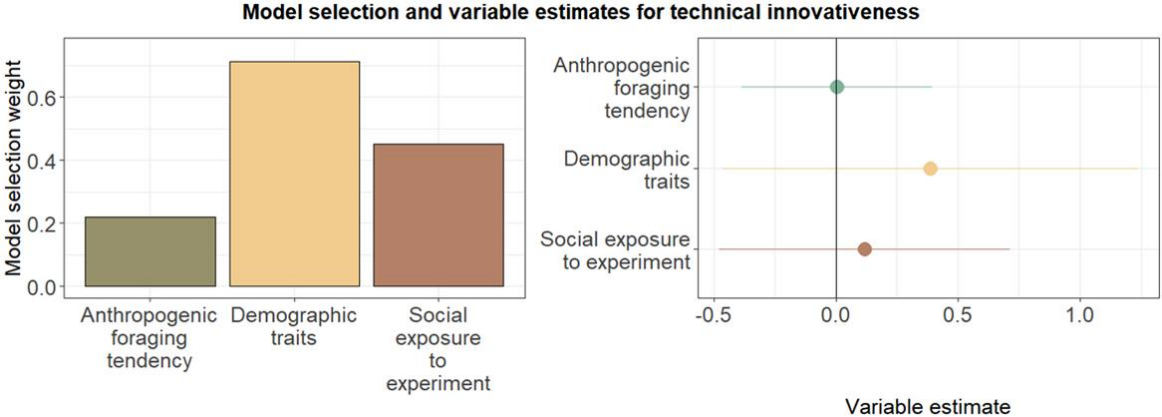
The probability that a monkey solved the foraging experiment through a technical innovation was more likely explained by demographic traits and previous social exposure to the experiment, rather than anthropogenic foraging tendency. (Left) Weights are calculated based on the sum of the AIC weights for every model that includes the respective dimension. (Right) The points indicate the effect estimate and the lines indicate the associated 95% confidence interval.

The tendency to switch between the lift and the pull options (phase 1) was not explained by demographic traits, previous social exposure, or a monkey's anthropogenic foraging tendency (*null* model weight: 0.64, Supplementary Material, Table S5). The ability of monkeys to innovate the technical solution was best explained by demographic and social exposure rather than the anthropogenic foraging tendency (Fig. 3, left). While the effect was largely uncertain (hence not significant, as expected given the reduced sample size), the demographic trait component showed the strongest effect, est. [CI_95%_] = 0.388 [−0.463, 1.239], followed by the social component, est. [CI_95%_] = 0.118 [−0.479, 0.715], and anthropogenic foraging tendency rates explained the least of the technical innovativeness scores, est. [CI_95%_] = −0.003 [−0.386, 0.392] (Fig. 3, right). Note that the *null* model (not accounting for any of these factors), was nonetheless not part of the 95% confidence set of the best models (Table 2), suggesting that these factors may collectively contribute to variation in innovativeness.

**Table 2:** Summary of the model selection approach.

Model names	K	AIC_C_	ΔAIC_C_	Log-likelihood	Weight	Cumulative weight
Demographic	2	39.78	0.00	−17.64	0.42	0.42
Social	2	41.14	1.35	−18.32	0.21	0.63
Demographic, Social	3	41.98	2.19	−17.47	0.14	0.77
Demographic, Anthropogenic Foraging Tendency	3	42.31	2.53	−17.64	0.12	0.89
Social, Anthropogenic Foraging Tendency	3	43.54	3.76	−18.25	0.06	0.95
Demographic, Social, Anthropogenic Foraging Tendency	4	44.74	4.96	−17.46	0.04	0.99
*null*	1	47.60	7.82	−22.74	0.01	1.00
Anthropogenic Foraging Tendency	2	49.37	9.59	−22.49	0.00	1.00

*K* represents the number of estimated parameters for each model, AIC_C_ is the Akaike Information Criterion corrected for small samples (Burnham et al. 2011), ΔAIC_C_ is the difference with the “best” model (demographic only model). Bold rows highlight models constituting the 95% confidence set.

### Absence of a behavioral flexibility syndrome

Monkeys that were the best innovators were also the ones most capable of performing a solution subsequently (beta-regression linking *IT* and *V*: est. [CI_95%_] = 1.688 [0.226, 3.150]; Supplementary Material, Table S12). Those innovators were not, however, the monkeys that switched between solutions the most (beta-regression linking *IT* and *SS*: est. [CI_95%_] = −0.863 [−2.577, 0.850]). In addition, switch tendency was unrelated to learning sensitivity (beta-regression linking *SS* and *V*: est. [CI_95%_] = −0.727 [−2.104, 0.651]).

### Innovativeness predicted problem-solving skills but not human food consumption

**Figure 4:**
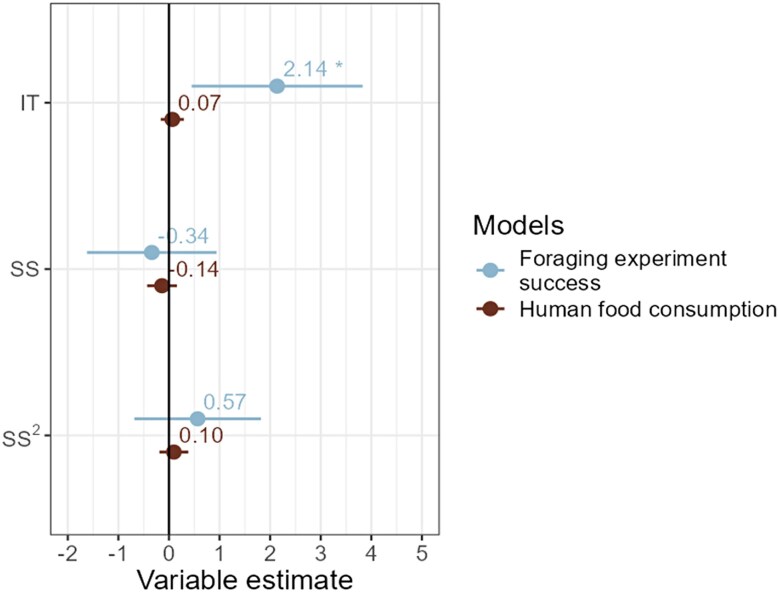
Forest plot of the beta-regression of individuals’ success in the foraging experiment and human food consumption as a function of the switch tendency between simple solutions (polynomial term, SS^2^ + SS) or the capacity of innovating a technical solution (IT). The points indicate the estimate, and the lines indicate the associated 95% confidence interval. The estimate values are given above each point and the “*” indicates the *P*-value significance.

Foraging experiment success did not correlate with human food consumption (ρ_pearson_ [CI_95%_] = 0.099 [−0.253, 0.428], *t*_31_ = 0.553, *P* = 0.584). *IT* significantly predicted monkeys’ performance in the foraging experiment (odds ratio [CI_95%_] = 2.14 [0.447, 3.83]; Table 3) but not *SS* ($\chi_{1}^{2}$ = 1.036, *P* = 0.596). Neither *IT*, *SS*, nor *SS*^2^ predicted an individual's human food consumption (*null* vs. *full* model comparison: Likelihood Ratio Test: $\chi_{3}^{2}$ = 1.828, *P* = 0.609; Table 3 and Fig. 4).

**Table 3:** Summary of success measure models.

	Foraging experiment success		Human food consumption	
Predictors	Estimates	CI_95%_	Estimates	CI_95%_
(Intercept)	1.00	0.65 to 1.55	0.03	0.03 to 0.04
*IT*	8.48	1.56 to 45.96	1.07	0.85 to 1.34
*SS*	0.71	0.20 to 2.56	0.87	0.65 to 1.17
*SS* ^2^	1.76	0.50 to 6.15	1.10	0.83 to 1.47
Observations	27		27	
*R* ^2^	0.284		0.069	

CI_95%_ = confidence interval at the 95% level. The estimates are raw estimates (ie, not adjusted for the logit transformation).

## Discussion

Our study investigated the link between anthropogenic foraging tendency and behavioral flexibility (switch tendency, innovativeness, and learning sensitivity) using a foraging experiment with semi-urban vervet monkeys. Participation in the task was high: over 80% of initial participants completed phase 1, providing a robust and representative sample of the population. We found, first, that monkeys with higher rates of anthropogenic foraging experience did not appear more behaviorally flexible, neither in their switch tendency, nor in their innovativeness. Second, the expected behavioral flexibility syndrome composed of these traits was not supported by our data. Third, behavioral flexibility did not predict monkeys’ human food consumption.

### Exaptation rather than adaptation

None of the 3 factors (anthropogenic foraging tendency, social exposure, or demographic traits) predicted switch tendency, and anthropogenic foraging tendency were less predictive of technical innovativeness than demographic traits or previous social exposure to the puzzle box (Table 2, Fig. 3). As anthropogenic foraging tendency was not a significant predictor of individuals’ behavioral flexibility, these results suggest that the traits observed in our foraging experiment may fall within the “latent solution” of cognitive capacities for vervet monkeys as a species (Tennie et al. 2020) rather than representing skills shaped by their urban experience. That is, the behavioral flexibility underpinning problem-solving may be species-wide, pre-existing traits evolved for different challenges, such as extractive foraging, rather than adaptations to urban life. Indeed, as dietary generalists, vervet monkeys benefit from their behavioral flexibility while foraging in natural environments where they switch between food items, eg from eating leaves up in trees to opening hard seedpods, licking nectar from flowers or rolling stones on the ground to access hidden insects.

Urban environments are often assumed to select for enhanced behavioral flexibility due to the presumed novelty of urban challenges (Sol et al. 2013). Since anthropogenic foraging tendency frequently involves the manipulation of human-made objects to access food, we expected that individuals who raid more would show higher levels of behavioral flexibility when interacting with our novel foraging task, but this was not the case. Our study population of vervet monkeys has experienced this semi-urban environment throughout multiple generations and certainly the entire lives of the participating monkeys. For these individuals, anthropogenic elements, such as human-made structures and objects like bins with lids do not represent novel challenges. This familiarity may alleviate the selection pressure for enhanced innovativeness. Instead, urban living may permit exaptation, where the expression of ancestral traits evolved for 1 function are co-opted for another (Johnson-Ulrich and Forss 2025), allowing urban monkeys to respond to familiar, albeit anthropomorphized, environments. Our empirical findings align with theoretical suggestions that urbanization does not necessarily require increased innovativeness, but rather a flexible application of pre-existing cognitive capacities such as the ability to learn about the urban environment (Leimar et al. 2023). Similar findings by Vardi and Berger-Tal (2022) show that behavioral flexibility of male house sparrows (*Passer domesticus*) is better predicted by the rate of anthropogenic change rather than its magnitude, suggesting that, in relatively stable urban environments, animals may not require novel cognitive adaptations to persist.

Experimental studies of cognitive traits in urban primates remain limited (Mangalam and Singh 2013; Roux et al. 2019; Pal et al. 2022; Ellington et al. 2024). Of these, only Pal et al. (2022) directly linked a metric of anthropogenic exposure to task performance in bonnet macaques (*Macaca radiata*) and found a positive correlation between locals and tourists actively feeding the monkeys and the frequency of monkey's sophisticated bottle opening techniques, although food provisioning may have confounded experience with motivation. In contrast, our data showed no such correlation, as anthropogenic foraging tendency did not predict innovativeness nor switch tendency. These differences highlight the possibility that these motivational differences also affect the interpretation of rural and urban population comparison studies. By examining individuals within the same population, we controlled for environmental differences in motivation, highlighting the value of within-population approaches when evaluating the effects of urban experience on cognition.

### Flexibility traits did not form a behavioral syndrome

Using a probabilistic modeling approach, we included the entire sequence of all 3 experimental phases and trials to trace an individual's behavioral actions and determined their individual switch tendency, innovativeness, and learning sensitivity. This provided a more robust reflection of the monkeys’ cognitive reasoning compared with trial averages or endpoint scores alone. Although previous work applying similar methods used binary outcomes and more trials (Lukas et al. 2024), our model validation (Supplementary Material, section S8) showed robust outcomes and a lack of sensitivity to arbitrary decisions we had to make.

Theory on the evolution of behavioral syndromes suggests that correlated sets of traits can evolve when feedback loops reinforce co-expression (Wolf and Weissing 2010). For example, in some species, boldness and aggression correlate because bolder individuals secure more resources, which further enhances aggressiveness (Wolf and Weissing 2010). If urban environments imposed such consistent selection on traits underpinning behavioral flexibility, we might expect stronger trait integration. Contrary to our predictions, we found no relationship between the 3 flexibility traits (switch tendency, innovativeness and learning sensitivity), except for a positive relationship between technical innovativeness and learning sensitivity (See Absence of a behavioral flexibility syndrome). Switch tendency was not associated with either of these. Both technical innovativeness and learning sensitivity are processes likely underpinning solving of more challenging physical problems (as posed in phase 2 and 3), while switch tendency possibly relates more to motor actions used by monkeys frequently, especially in the urban habitat. This suggests that switch tendency may reflect an exploratory tendency or motivational trait, rather than an advanced cognitive capacity. These findings are consistent with prior work showing that behavioral flexibility measured by switching between options was not correlated with learning in gray squirrels (Chow et al. 2016).

The absence of an adaptive, behaviorally flexible syndrome suggests that selection may not uniformly act upon switch tendency, innovativeness, and learning sensitivity. However, there may be adaptive value in maintaining trait variation within a population. Just as theoretical modeled mixed-ability groups (eg, diverse problem-solving agents) can outperform homogenous groups of the high-ability problem solvers (Hong and Page 2004), variation in motivational traits and flexibility among group members may better buffer populations against a range of urban challenges. Beyond improved behavioral flexibility, traits in vervet monkeys such as boldness or risk-taking may interact with, or even overshadow, behavioral flexibility in determining urban success, further contributing to the maintenance of trait variation within the group.

### Flexibility traits relating to problem-solving are not related to human food consumption

Innovativeness, but not switch tendency, significantly predicted individual success in the foraging experiment (Table 3, Fig. 4), which aligns with comparative findings across bird species (Audet et al. 2024). Conversely, in our study, neither trait predicted an individual's consumption of anthropogenic food sources. This was unexpected, given that human food consumption represents attractive, opportunistic, high calorie foraging opportunities that benefit individuals (at least in the short term), and during which monkeys encounter human artifacts and as such occasionally may need to innovatively solve technical problems. However, at our study site of Simbithi Eco-Estate, vervet monkeys still forage primarily on natural vegetation (Thatcher et al. 2020). Therefore, the vast abundance of forest patches likely buffers any potential effects of urbanization on the need to exploit human food sources, and, consequently, diminishing their necessity for technical innovativeness (Gallo et al. 2017).

Since it is difficult to obtain classic fitness measures to quantify success in this environment, such as longevity and reproductive success directly in long-lived species like primates (Stearns 2000; Knapp et al. 2021), we used human food consumption as an approximation of success in habitat exploitation. However, this proxy did not relate to any of our behavioral flexibility measures. Foraging success, especially risky ones involving entering human houses, may be mediated by personality traits or social factors. For instance, dominant individuals may monopolize human retrieved food or benefit through sharing from other bold individuals’ efforts to take such risks (Pansini 2011; Marty et al. 2020). Additionally, vervet monkeys are known to socially learn their dietary preferences (van de Waal et al. 2013, 2014), which may further reduce selection on individual flexibility and contribute to the observed missing link between innovation and human food consumption in our data. Consequently, social relationships may be more decisive for food tolerance and the development of food preferences and foraging skills. To sum up, our results suggest that behavioral flexibility, while beneficial in a controlled foraging experiment, is likely not decisive of success in semi-urban environments.

### Limitations and broader implications

Although our results suggest that current levels of behavioral flexibility in vervet monkeys suffice for them to thrive in the current anthropogenic environment, our measurements represent only a snapshot in time, and they do not rule out future adaptative change. While most features specific to the anthropogenic habitat are independent from the monkeys’ presence, the human inhabitants are often using targeted prevention measures to hinder monkeys entering structures and trash bins (ie, monkey screens to prevent access to houses, bin clips, spinning bin tops). As such, monkeys may be involved in an arms race with humans (as observed in cockatoos (*Cacatua galerita*) interacting with human deterrents: Klump et al. 2022), applying selective pressures affecting monkeys’ cognitive profiles. As Simbithi Eco-Estate was established in 2003, the selection pressures may change as prevention methods increase and the eco-estate matures, potentially leading to increased human-wildlife conflicts (Barrett et al. 2019). Re-testing this population of monkeys after several years may reveal changes in the population's behavioral flexibility. Combining this with the known life-histories and pedigrees of the monkeys in the future will lead to a better understanding of how the urban environment shapes animal cognition and may help develop management strategies to limit human-vervet conflicts.

## Supplementary Material

arag070_Supplementary_Data

## Data Availability

Analyses reported in this article can be reproduced using the data provided by Barnes et al. (2026). All data is available on figshare (https://figshare.com/s/3d508e0de03b9377d102).
